# Efficacy of Vitamin D Supplementation in Multiple Sclerosis (EVIDIMS Trial): study protocol for a randomized controlled trial

**DOI:** 10.1186/1745-6215-13-15

**Published:** 2012-02-08

**Authors:** Jan Dörr, Stephanie Ohlraun, Horst Skarabis, Friedemann Paul

**Affiliations:** 1 NeuroCure Clinical Research Center, Charité - Universitätsmedizin Berlin, Berlin, Germany; 2 Clinical and Experimental Multiple Sclerosis Research Center, Charité - Universitätsmedizin Berlin, Berlin, Germany; 3 Freelance biometrician and statistical consultant (professor emeritus), Gross Oesingen, Germany; 4 Experimental and Clinical Research Center, Max Delbrueck Center for Molecular Medicine and Charité - Universitätsmedizin Berlin, Berlin, Germany

**Keywords:** multiple sclerosis, vitamin D, cholecalciferol, clinical trial, intervention, immunomodulation, interferon-β.

## Abstract

**Background:**

Multiple sclerosis is the most common chronic inflammatory disease of the central nervous system in young adults. Despite the fact that numerous lines of evidence link both the risk of disease development and the disease course to the serum level of 25-hydroxyvitamin D it still remains elusive whether multiple sclerosis patients benefit from boosting the serum level of 25-hydroxyvitamin D, mainly because interventional clinical trials that directly address the therapeutic effects of vitamin D in multiple sclerosis are sparse. We here present the protocol of an interventional clinical phase II study to test the hypothesis, that high-dose vitamin D supplementation of multiple sclerosis patients is safe and superior to low-dose supplementation with respect to beneficial therapeutic effects.

**Methods/Design:**

The EVIDIMS trial is a German multi-center, stratified, randomized, controlled and double-blind clinical phase II pilot study. Eighty patients with the diagnosis of definite multiple sclerosis or clinically isolated syndrome who are on a stable immunomodulatory treatment with interferon-β1b will be randomized to additionally receive either high-dose (average daily dose 10.200 IU) or low-dose (average daily dose 200 IU) cholecalciferol for a total period of 18 months. The primary outcome measure is the number of new lesions detected on T2-weighted cranial MRI at 3 tesla. Secondary endpoints include additional magnetic resonance imaging and optical coherence tomography parameters for neuroinflammation and -degeneration, clinical parameters for disease activity, as well as cognition, fatigue, depression, and quality of life. Safety and tolerability of high-dose vitamin D supplementation are further outcome parameters.

**Discussion:**

In light of the discrepancy between existing epidemiological and preclinical data on the one hand and available clinical data on the other the EVIDIMS trial will substantially contribute to the evaluation of the efficacy of high-dose vitamin D supplementation in MS patients. The study design presented here fulfills the criteria of a high-quality clinical phase II trial in MS.

**Trial Registration:**

ClinicalTrials.gov Identifier: NCT01440062

## Background

Multiple sclerosis (MS) is the most common chronic inflammatory disease of the central nervous system (CNS) in western countries [1]. Based on autoimmune mediated damage to both glial and neuroaxonal structures, the disease is not yet curable and therapeutic options mainly focus on the control of the autoimmune reaction. The approved therapies include immunomodulatory (interferon-β, glatiramer acetate), (selective) immunosuppressive (fingolimod, azathioprine, mitoxantrone) or antibody-mediated (natalizumab) approaches. Additionally, new drugs are currently under investigation at different clinical stages. Although efficacy of all these drugs has been proven in large-scale clinical trials, some major issues still remain unsolved including partial efficacy, inconvenience of parenteral application, safety and tolerability aspects, insufficient neuroprotective capacity, and last but not least considerable treatment costs [2,3]. Against this background the need for an improvement of therapeutic options in MS remains.

Numerous lines of evidence link both the risk of disease development and the disease course to the serum level of 25-hydroxyvitamin D which is the biologically inactive storage form of vitamin D (extensively reviewed in [4]): it is long known that the geographic distribution of MS prevalence depends on latitude which is associated with both sunlight intensity and vitamin D serum levels [5-8]. Particularly, increased sun exposure during childhood has been consistently shown to decrease the risk of MS [9-11]. Several epidemiological studies provide a direct correlation between vitamin D intake and vitamin D serum levels with both the risk for new disease development and the course of already established MS [12-15]. Further evidence for a beneficial role of vitamin D derives from the experimental autoimmune encephalomyelitis (EAE), which is the best established animal model for MS: it has been demonstrated that treatment with 1,25-dihydroxyvitamin D, the biologically active form of vitamin D, prevents the development of murine EAE (preventive approach) [16,17] and, commenced at onset of symptoms, reversibly blocks the progression of disease (therapeutic approach) [17]. Importantly, a recent EAE study provided evidence for a synergistic effect of a vitamin D analogue and interferon-β [18]. Less clear are the mechanisms of action by which vitamin D exerts its beneficial effects in autoimmune CNS disease. Modulatory effects on cellular and soluble components of the underlying immune reaction are probably most important [4,19]. Additionally, prevention of demyelination has also been demonstrated recently in a model of toxic demyelination [20]. Compared to the large body of existing preclinical data, data on treatment effects derived from well-designed clinical trials are scarce, and most clinical trials so far focused rather on safety and tolerability aspects. Weekly peak doses of up to 280.000 IU 25-hydroxyvitamin D and long term treatment with a mean weekly dose of at least 70.000 IU for 36 weeks have been well-tolerated. In particular, there was no evidence for treatment-related hypercalcaemia [21,22]. According to a review on the safety of high-dose vitamin D supplementation, daily doses of at least 10.000 IU can be considered safe in individuals with normal renal function [23]. Less clear is the optimal serum level for immunomodulatory treatment effects and hence the optimal daily dose [24]. A trend for a reduction of the annualized relapse rate was observed in clinical trials with daily doses of 5.000 to 10.000 IU, although these studies were not powered to detect a treatment effect on clinical parameters [22,25]. The effect on MRI parameters is even more obscure. Accordingly, a recent Cochrane review concluded that "multi-centered RCTs (randomized controlled trials) with a focus on clinical as well as immunological and MRI outcomes (...) are still required" [26].

Against this background, we here present the study protocol for a randomized controlled clinical phase II pilot study on the effect of vitamin D supplementation on MRI in patients with relapsing-remitting MS or clinically isolated syndrome (CIS). We hypothesize, that high-dose supplementation is superior to low-dose supplementation with respect to imaging parameters including MRI and optical coherence tomography (OCT) as well as clinical parameters.

## Methods/Design

### Trial design

EVIDIMS is a national, multi-center, stratified, randomized, controlled, and double-blind clinical phase II trial conducted at approximately ten sites located in or close to Berlin, Germany. Recruitment will start in 12/2011. Eighty patients with relapsing-remitting MS or CIS who are on a stable immunomodulatory treatment with interferon-β1b will be randomized to either high-dose (20.400 IU) or low-dose (400 IU) 25-hydroxyvitamin D supplementation to be taken every other day for 18 months add-on to interferon-β1b. The detailed study design is provided below according to the revised CONSORT statement [27,28]. The study is approved by the local ethics committee and the German Competent Authority (Federal Institute for Drugs and Medical Devices) and is registered at both the European Union Drug Regulating Authorities (Eudra-CT: 2011-002785-20) and Clinicaltrials.gov (NCT01440062). The study will be conducted in accordance to the Declaration of Helsinki in its currently applicable version, the guidelines of the International Conference on Harmonization of Good Clinical Practice (ICH-GCP), and the applicable German laws. All participants will be required to give informed written consent. The trial will be monitored according to ICH-GCP.

### Participants

Inclusion criteria for participation in the EVIDIMS trial comprise the diagnosis of either definite relapsing-remitting MS according to the revised 2005 McDonald criteria [29] or CIS, an age of 18 to 65 years, a score of less than 6.5 on the Expanded Disability Status Scale (EDSS) [30], and being relapse-free for at least 30 days. All participants are required to be on a stable immunomodulatory treatment regimen with interferon-β1b (8 MIU, every other day) for at least three months. In women of childbearing potential highly effective methods of birth control defined as a contraception method with a PEARL-index < 1 and a negative pregnancy test at screening is mandatory. The main exclusion criteria include any other disease course than relapsing-remitting MS or CIS, any disease other than MS that better explains the symptoms and signs, any immunomodulatory or immunosuppressive treatment other than interferon-β1b within the preceding three months, intake of vitamin D containing products within in the preceding six months, participation in any other interventional clinical trial in the last three months, pregnancy or breast feeding. Additionally, participants may not have relevant liver, kidney or bone marrow dysfunction or suffer from sarcoidosis, nephrolithiasis, or hypercalcaemia. Concomitant medication with hydrochlorothiazide, barbiturates, phenytoin, digitalis or other vitamin D containing products is not allowed. To ensure homogeneity in terms of sunlight intensity and duration as a possible confounder, patients will be recruited from a single geographic region (in or close to Berlin, Germany).

### Randomization and interventions

Eligibility of patients will be determined at the screening visit. At baseline visit, patients who qualified for participation in the study will be stratified according to gender, disease course, and 25-hydroxyvitamin D serum level at screening and then randomized 1:1 into two intervention groups. Group A will receive 20.400 IU cholecalciferol orally every other day; group B will receive 400 IU cholecalciferol orally every other day, which corresponds to the daily dose recommended by the German Nutrition Society http://www.dge.de. In both arms, vitamin D treatment will be add-on to a stable immunomodulatory treatment regimen with interferon-β1b. Regular study visits are carried out after 1, 3, 6, 9, 12, and 18 months. Investigations to be performed on the respective study visits are displayed in table 1. An optional follow-up visit will be offered 24 months after randomization. If necessary, additional unscheduled visits can be performed at any time.

**Table 1:** Time schedule of regular study visits and outcome parameters

	Screening (Week -2)	V0 (Baseline) ± 7 days	V1(Month 1) ± 7 days	V2 (Month 3) ± 7 days	V3 (Month 6) ± 14 days	V4 (Month 9) ± 14 days	V5 (Month 12) ± 14 days	V6 (Month 18) ± 21 days	Follow up (Month 24) ± 8 weeks (optional)
Informed consent	*√*								

In/exclusion criteria	*√*	*√*							

Randomization		*√*							

Evaluation of relapses	*√*	*√*	*√*	*√*	*√*	*√*	*√*	*√*	*√*

Concomitant medication	*√*	*√*	*√*	*√*	*√*	*√*	*√*	*√*	*√*

Physical examination	*√*				*√*		*√*	*√*	

Vital signs	*√*	*√*	*√*	*√*	*√*	*√*	*√*	*√*	*√*

MSFC	*√*				*√*		*√*	*√*	*√*

EDSS	*√*				*√*		*√*	*√*	*√*

ECG	*√*							*√*	

Safety lab	*√*	*√*	*√*	*√*	*√*	*√*	*√*	*√*	

Immunology blood sampling	*√*				*√*		*√*	*√*	

MRI	*√*						*√*	*√*	*√*

OCT	*√*						*√*	*√*	*√*

Visual contrast parameters	*√*						*√*	*√*	*√*

Quantitative motor function tests	*√*						*√*	*√*	*√*

Questionnaires (fatigue, depression, QoL)	*√*				*√*		*√*	*√*	*√*

Cognitive testing (SDMT, FST)	*√*							*√*	*√*

Drug account			*√*	*√*	*√*	*√*	*√*	*√*	

Adverse event reporting			*√*	*√*	*√*	*√*	*√*	*√*	

### Outcome parameters

The primary endpoint is the cumulative number of new hyperintense lesions on cranial T2-weighted MRI during the treatment period of 18 months. Additional secondary MRI endpoints comprise number and volume of T1-hypointense and T2-hyperintense lesions, proportion of patients without any new T1-hypointense and T2-hyperintense lesions, number of contrast enhancing lesions, brain atrophy as determined by normalized brain volumetry, and changes in brain metabolism as determined by magnetic resonance spectroscopy (MRS). All MRI/MRS investigations will be performed on a 3 tesla MRI scanner (Siemens, Germany). All MRI parameters will be assessed by an experienced evaluator who is blinded to both clinical data and treatment allocation. Secondary clinical endpoints include the annualized relapse rate and the proportion of relapse-free patients. A relapse is defined as de novo development or aggravation or re-occurrence of a preexisting neurological abnormality compatible with MS which lasts a minimum of 24 hours, is separated by at least 30 days from a preceding clinical event and does not occur in the context of fever. Additional secondary endpoints comprise the disease progression as determined by EDSS (evaluated by an independent neurologist), Multiple Sclerosis Functional Composite (MSFC), cognition as determined by the Faces Symbol Test (FST) and the Symbol Digit Modalities Test (SDMT), fatigue and depression as well as quantitative assessment of motor function [31,32]. Additional parameters include retinal nerve fiber layer thickness and macular volume which are increasingly recognized as markers for disease progression in MS [33], visual contrast sensitivity and low contrast visual acuity, 25-hydroxyvitamin D serum levels, and quality of life. Assessment of all endpoints will be done by experienced personnel blinded to both the clinical data and treatment allocation. Peripheral venous blood will be sampled prior and during the intervention for evaluating the effect on cellular and soluble components of the immune system. Safety and tolerability will be assessed by vital signs, safety lab, ECG, and adverse event reporting. The time schedule of all regular evaluations is displayed in table 1.

### Sample size

Since available data on the effect of vitamin D on the development of new T2-hyperintense lesions on cranial MRI are not sufficient for an exact statistical sample size calculation the study is designed as pilot study with an *a priori *determined sample size of 40 patients per intervention arm, in total 80 patients. Based on this sample size, a mean reduction of 1.5 new T2-hyperintense lesions in the high-dose treatment compared to the low-dose regimen will be detected with a power of 0.83.

### Blinding

Both, patients and the entire study staff will remain blinded throughout the complete treatment period of 18 months. Treatment allocations will only be disclosed after the final database lock. Substantial efforts are made to maintain blinding: Since high-dose formulation (oil) does not match the low-dose formulation (tablet), patients in the high-dose arm will take 400 IU tablets in addition to 20.000 IU oil, whereas patients in the low-dose arm will receive an identical volume of placebo-oil in addition to a 400 IU tablet. The patients are primarily handled by a treating physician who is in charge of the evaluation of inclusion or exclusion criteria, adverse events, relapses, side effects, etc. All neurological examinations will be done by an independent evaluating physician. Accordingly, evaluation of all other paraclinical parameters including MRI and OCT will be done by independent examiners. Any patient-related crosstalk between treating and evaluating physicians or examiners is prohibited unless required because of safety concerns. An easy unblinding procedure allows for rapid unblinding of a patient in case of a medical necessity. Unblinding inevitably results in the exclusion of the respective patient.

### Statistical methods

Evaluation of endpoints will be carried out by both intention-to-treat and per-protocol analyses. Statistical tests and presentation will be appropriate to the category and distribution of the respective variables. Analysis of OCT data will be performed by statistical models which account for intra-patient inter-eye relations. The test level for statistical significance of differences between both treatment arms is defined as p = 0.05 for all tests.

### Funding

The EVIDIMS trial is funded in part by a limited grant of Bayer Vital GmbH, Germany and supported by the German Research Foundation (DFG Exc 257).

## Discussion

In light of the discrepancy between existing epidemiological and preclinical data on the one hand and available clinical data on the other the study design presented here has the potential to substantially contribute to the evaluation of the efficacy of vitamin D supplementation in MS and CIS patients. The randomized, controlled, double-blinded study design and the implementation of independent evaluation of outcome parameters fulfill the criteria for a high-quality clinical phase II trial in MS [34]. Some aspects of the study design however may deserve a closer discussion: Why was an active treatment regimen instead of a placebo treatment chosen for the control arm, i.e. low-dose vitamin D? In fact, this question was heavily debated. Although we do not know whether this represents rather a causal factor or a consequence, serum levels of 25-hydroxyvitamin D are often quite low in MS patients [13,35]. Thus, we expect that low serum levels or even vitamin D deficiency will be detected in a substantial number of screened study candidates. From an ethical point of view and bearing in mind the importance of vitamin D for bone metabolism it would be difficult not to supplement these patients with vitamin D. On the other hand, the daily dose provided in the control arm may not be immunologically active itself as this would prevent the detection of any difference between both groups. Thus, the daily dose recommended by the German Nutrition Society for this group of age which corresponds to 5 μg or 200 IU and which most probably has no immunomodulatory potential represents a compromise between ethical concerns and efficacy aspects. Another important question relates to the dose used in the high-dose arm. In fact, we do not know, at which minimum doses or serum levels vitamin D starts to have immunomodulatory effects. To prevent failure of the trial because of an insufficient treatment dose, we choose the maximum dose for which sufficient safety data are available, which currently corresponds to 10.000 IU per day [23]. It might well be that already smaller doses would be sufficient, but on the other hand it is rather unlikely, that if this dose does not demonstrate any treatment effect, even higher daily doses would do. A further question might be why an add-on regimen to an established immunomodulatory treatment with interferon-β was chosen? In fact, from a methodological point of view, a monotherapeutic design would be preferable. However, since disease-modifying drugs are established and approved for the treatment of MS it would again be unethical to withhold these treatment options in favor of an experimental approach. The restriction to interferon-β as immunomodulatory treatment is explained by the need for maximum homogeneity in the trial cohort on the one hand and the reported synergistic effects of interferon-β and the vitamin D system on the other [18]. Finally, one might suspect that in view of the rather small sample size of 80 participants the study might be underpowered to detect a significant difference between both groups. However, the study will be able to detect a difference in mean new T2-hyperintense lesions per year of 1.5 with a power of more than 0.8. In the pivotal interferon-β1b trial, verum treatment resulted in a reduction of 2.0 lesions per year as compared to a placebo arm [36]. Thus, if high-dose supplementation with vitamin D is indeed effective, a reduction of 1.5 lesions per year would not be unrealistic.

In conclusion, vitamin D has the potential for a safe, orally available and cheap treatment option in MS and the EVIDIMS trial may help to close the existing gap between available promising preclinical and the lacking clinical data on the immunomodulatory efficacy of vitamin D in MS.

### Trial status

The EVIDIMS trial is currently recruiting patients.

## List of abbreviations

CIS: Clinically isolated syndrome; CNS: Central nervous system; ECG: Electrocardiogram; EDSS: Expanded disability status scale; EVIDIMS: Efficacy of Vitamin D Supplementation in Multiple Sclerosis; FST: Faces symbol test; ICH-GCP: International Conference on Harmonization of Good Clinical Practice; IU: International units; MRI: Magnetic resonance imaging; MRS: Magnetic resonance spectroscopy; MS: Multiple sclerosis; MSFC: Multiple sclerosis functional composite; OCT: Optical coherence tomography; QoL: Quality of life; SDMT: Symbol digit modalities test.

## Competing interests

FP received travel support and speaker honoraria by Bayer Vital GmbH. JD received speaker honoraria by Bayer Vital GmbH. The authors declare that they have no competing interests. The partial funder of the study (Bayer Vital GmbH, Germany) is neither involved in the study design nor the interpretation of data or publication of results.

## Authors' contributions

JD designed the study, drafted the study protocol and drafted the manuscript. SO was in charge of all regulatory affairs and critically revised both, the study protocol and manuscript. HS is the biometrician of the study and critically revised both, the study protocol and manuscript. FP designed the study and also critically revised both, the study protocol and manuscript. All authors have given final approval of the version to be published.
